# Clinical Feature and Outcome of Childhood Status Epilepticus in a Teaching Hospital, Odisha, India

**DOI:** 10.7759/cureus.10927

**Published:** 2020-10-13

**Authors:** Kedarnath Das, Santosh K Das, Sarbeswar Pradhan, Priyadarshini I Sahoo, Nirmal K Mohakud, Arakhita Swain, Saroj Satpathy

**Affiliations:** 1 Pediatrics, Srirama Chandra Bhanja Medical College and Hospital, Cuttack, IND; 2 Pediatrics, Kalinga Institute of Medical Sciences, Bhubaneswar, IND; 3 Medicine, Srirama Chandra Bhanja Medical College and Hospital, Cuttack, IND

**Keywords:** convulsion, seizure, morbidity and mortality, antiepileptic drugs, status epilepticus, etiology

## Abstract

Objectives

The main aim is to find out the clinical feature and outcome of status epilepticus (SE) in children managed in a teaching hospital. The secondary aim is to identify the risk factors influencing the adverse outcomes.

Methods

In this prospective cohort, children aged 1 month to 14 years with SE as per the International League Against Epilepsy’s new guideline (2016) who presented to the emergency department during the period of November 2017 to October 2019 were enrolled. Clinical profile, treatment, and outcome of cases (n = 94) were noted.

Results

The majority of children, 60 (63.82%), were less than five years of age. Prior history of seizures was present in 33 (35.1%) cases, whereas 61 (64.9%) cases presented with SE as the first episode of seizure. In 14 (42.4%) previous seizure cases, SE was due to drug default. No response to first-line antiepileptic drug (AED) was seen in 84 (89.37%) cases. Acute symptomatic etiology was the commonest etiology of SE in 64 (68%) cases, of which neuro-infections accounted for 44 (46.80%) cases. Longer duration (>60 minutes) of status (p < 0.01), ventilator support (p < 0.0001), and circulatory impairment (p < 0.0001) were attributable risk factors for mortality. A total of 28 children died (mortality rate, 29.8%), and 11 showed the persistence of their neuro-deficit.

Conclusions

Neuro-infection is the most common etiology of SE in children. Longer duration of SE, more lag time for receiving the first AED, respiratory failure, and presence of shock are independent predictors for poor outcome. Hence, cessation of convulsion at the earliest leads to improved outcomes.

## Introduction

Status epilepticus (SE) is a medical emergency, and its neurological outcome is a concern to every pediatrician in developing countries. The estimated convulsive status epilepticus (CSE) prevalence is 14.5 per 100,000 per year in developed countries [1], but population-based studies are not available from developing countries. There is significant morbidity (28‐34%) and mortality (7-22%) with SE despite advancement in treatment protocols in the last decade [2,3]. It is documented that if convulsion persisted beyond 10 minutes, it leads to irreparable brain damage and difficulty in controlling the seizure [4]. Childhood survivors of SE may develop long-term consequences such as developmental delay, cognitive impairment, and recurrent seizure. Therefore, it is of paramount importance to control seizure activity within 5-10 minutes to prevent neuronal damage. Benzodiazepines (BZD) are the first-line drugs to control seizure within 5-10 minutes of convulsion [5,6]. It should be followed by second-line drugs in the next 10-20 minutes. If the convulsion does not get control, third-line drugs such as anesthetic medicines through continuous infusion should be given within 30-60 minutes [6].

Previous studies have described the clinical manifestation and outcomes of SE taking 30 minutes as a cut-off seizure duration [2,7]. Presently, SE is defined by the International League Against Epilepsy (ILAE) as any active seizure of ≥5 minutes’ duration or recurrent episodes of seizures without gaining consciousness in between the seizure episodes [8]. Using this definition, Chetan et al. [3] provided important information regarding etiological profile, response to various antiepileptic drugs (AEDs), and immediate outcome of SE in a prospective study from India. But data regarding clinical profile, predictors of morbidity, and mortality using prospective studies are limited with the current definition of SE from Eastern India.

The objective of the study is to determine the clinical features, etiology, response to treatment with BZD, and immediate outcome, and to identify the risk factors influencing adverse outcomes in children with SE admitted to the pediatric ward/ intensive care unit in a referral teaching hospital.

## Materials and methods

This prospective observational study included children between 1 month and 14 years of age admitted in the pediatrics ward of S. C. B. Medical College situated in a tertiary care teaching hospital from Eastern India over a two-year period (November 2017 to October 2019). Approval of the Institutional Ethical Committee was obtained before initiation of the study. During this period, 94 children with SE as per ILAE’s new guideline (2016) [8] were admitted. SE was defined as active seizures of ≥5 minutes’ duration or recurrent episodes of seizures without gaining consciousness in between the seizure episodes [5]. Informed consent was obtained from parents or guardians of the included children. The total duration of seizure was obtained from the patient’s mother or guardian, and other records of the referring doctor. Once the child was stabilized, data regarding age, sex, duration of seizures before and after admission, type and number of AEDs used for control, history of previous seizure pattern, adherence to treatment, perinatal history, developmental history, family history, and history of coexisting medical conditions were recorded. A general physical and detailed neurological examination was performed. Investigations, such as complete blood count, serum calcium, random blood sugar, serum sodium, urea and creatinine, neuroimaging, CSF (cerebrospinal fluid) examination, and IgM (immunoglobulin M) enzyme-linked immunosorbent assay for scrub typhus, were performed. Electroencephalography (EEG) was performed to ascertain etiology and guide treatment. During the hospital stay, recurrence of seizures, the subsequent need for intubation and mechanical ventilation, and days spent in the pediatric ICU were recorded. Detailed treatment, response, and outcome were also noted.

Type and etiology of SE were classified based on the SE classification report of the ILAE Task Force [8]. Refractory status epilepticus (RSE) was defined as the persistence of seizures in spite of using two appropriate anticonvulsants at therapeutic doses, with a minimum duration of status of 60 minutes (with observation and history). Though febrile seizures are a part of acute symptomatic etiology, it has been considered separately for analysis to avoid dilution of the severity of acute neurological insults.

Data were analyzed using SPSS Version 21 (IBM Corp., Armonk, NY, USA). A p-value of less than 0.05 was considered statistically significant. Furthermore, univariate analysis was used to find out the significance of various parameters in the outcome of the present study.

## Results

Of the total 94 children studied, the median (IQR) age was 3 (1-7) years. The majority of children 60 (63.82%) were less than five years of age. There was a male predominance (61 [64.9%]). Out of hospital seizure onset was present in 82 (87.2%) cases. A total of 49 (59.7%) patients received pre-hospital treatment with diazepam (30) and phenytoin/phenobarbitone (19). Fever was present in 63 (67%) cases before the onset of seizures. Prior history of seizures was present in 33 (35.1%) cases, whereas the first episode of seizure with 61 (64.9%) cases. Most of the children, 46 (48.9%), had an SE duration of <30 minutes. The seizure was terminated after administration of BZD in 10 (10.63%) cases, but second- and/or third-line drugs were needed in 84 (89.37%) cases. However, 27 (28.72%) patients progressed to RSE. Generalized seizures, mostly generalized tonic-clonic (GTC) seizures, were observed in 87 (92.6%) children. Acute symptomatic etiology was the most common cause of SE in 64 (68%) cases. Neuro-infections accounted for 44 (46.80%) cases and febrile seizures for 5 (5.3%) cases. Again, acute symptomatic etiologies were the major causes of SE in children less than five years of age, whereas remote symptomatic causes were more in children aged 3-10 years (Table 1).

**Table 1:** Clinical and etiological features of SE children (n = 94) SE, status epilepticus

Characteristics	No (%)
Median age, in years	3 (1-14)
Less than 5 years	60 (63.82)
Male	61 (64.9)
History of fever before the onset	63 (67)
Patients received pre-hospital treatment	49 (59.7)
First episode of seizure	61 (64.9)
Prior history of seizures	33 (35.1)
Duration of SE	
<30 minutes	46 (48.9)
30-60 minutes	4 (4.3)
>60 minutes	44 (46.8)
Seizure type	
Focal	7 (7.4)
Generalized	87 (92.6)
Etiology	
Acute symptomatic	64 (68%)
Viral encephalitis	24 (25.5)
Pyogenic meningitis	14 (14.9)
Tubercular meningitis	3 ( 3.2)
Metabolic	7 ( 7.4)
Hypertensive encephalopathy	4 ( 4.3)
Intra-cranial hemorrhage	2 ( 2.1)
Febrile seizure	5 ( 5.3)
Scrub encephalitis	3 ( 3.2)
Remote symptomatic	17 (18.1)
Cerebral palsy	12 (12.8)
Post-encephalitis hydrocephalus	5 (5.3)
Progressive	1 (1.1)
Cryptogenic	12 (12.8)

As shown in Table 2, the overall mortality was 28 (29.78%). The acute symptomatic group had the highest mortality of 20 (71.42%). Neurological deficit persisted in 11 (11.70%) cases.

**Table 2:** Treatment and outcome of status epilepticus in children Morbidity: after treatment of SE, patients who developed focal neurological deficits and neuro-cognitive impairment FIRES, Febrile Infection-Related Epilepsy Syndrome

Etiology	No. of cases	Death	Morbidity	Recovery
Acute symptomatic	64 (68)	20 (71.42)	8 (72.72)	36 (65.45)
Viral encephalitis	24	8	1	15
Pyogenic meningitis	14	5	0	9
Tubercular meningitis	3	1	2	0
Metabolic	7	3	1	3
Hypertensive encephalopathy	4	2	1	1
Intra-cranial hemorrhage	2	0	1	1
FIRES	1	0	1	0
Stroke	1	0	1	0
Febrile seizure	5	1	0	4
Scrub encephalitis	3	0	0	3
Remote symptomatic	17 (18.1%)	5 (29.41)	1 (9.09%)	11 (20%)
Cerebral palsy and post-encephalitis sequelae	12	2	1	9
Hydrocephalus	5	3	0	2
Progressive neurological disease	1 (1.1%)	1 (100)	0	0
Cryptogenic	12 (12.76%)	2 (16.67)	2 (18.18%)	8 (14.54%)
Total	94 (100%)	28 (29.78)	11 (11.70%)	55 (58.51%)

On analyzing the major contributing risk factors for mortality, duration of CSE > 60 minutes had higher mortality (n = 21 [80.8%]) compared to CSE <30 minutes (n = 3 [5.1%]) (p < 0.01).The fatality in children with RSE was 85.2% compared to non-RSE 7.5% (p < 0.0001). Ventilator support (p = 0.0001) and circulatory impairment (p = 0.0001) were the important attributable risk factors for mortality (Table 3).

**Table 3:** Analysis of risk factors for mortality in children with status epilepticus GTCS, generalized tonic-clonic seizure; AED, antiepileptic drug; RSE, resistant status epilepticus; NRSE, non-resistant status epilepticus; CSE, convulsive status epilepticus

Risk factor	Mortality (%)	No mortality (%)	𝟀^2^ value	p-Value
Age				
<1 year	7 (24.1)	22 (75.9)	4.83	0.18
1-5 years	8 (25.8)	23 (74.2)		
5-10 years	6 (26.1)	17 (73.9)		
>10 years	7 (63.6)	4 (36.4)		
Seizure episode				
First episode	19 (31.1)	42 (68.9)	0.15	0.69
Prior history of seizure	9 (27.3)	24 (72.7)		
Seizure types				
Secondary generalized	2 (28.6)	5 (71.4)	1.80	0.61
Generalized clonic	0 (0)	1 (100)		
Generalized tonic	0 (0)	6 (100)		
GTCS	26 (32.5)	54 (67.5)		
Lag time for receiving first AED				
<30 minutes	3 (5.1)	56 (94.9)	46.33	0.0001
30-60 minutes	4 (44.4)	5 (55.6)		
>60 minutes	21 (80.8)	5 (19.2)		
Response to treatment				
RSE	23 (85.2)	4 (14.8)	55.58	0.0001
NRSE	5 (7.5)	62 (92.5)		
Total duration of CSE				
<30 minutes	1 (2.2)	45 (97.8)	29.69	<0.01
30-60 minutes	2 (50.0)	2 (50.0)		
>60 minutes	25 (56.8)	19 (43.2)		
Pre-existing neurological deficit				
Present	7 (35.0)	13 (65.0)	0.33	0.56
Absent	21 (28.4)	53 (71.6)		
Ventilator				
Yes	22 (88.0)	3 (12.0)	55.18	0.0001
No	6 (8.7)	63 (91.3)		
Shock				
Yes	16 (76.2)	5 (23.8)	27.84	0.0001
No	12 (16.4)	61 (83.6)		
Etiology				
Acute symptomatic (except febrile seizure)	19 (32.2)	40 (67.8)	0.44	0.506
Other etiology	9 (25.7)	26 (74.3)		

## Discussion

This prospective study elucidates the clinical outcome and response to treatment in childhood SE in a tertiary care teaching institute of a developing country. Taking into account the ILAE 2016 guideline, it is found that acute symptomatic etiology, mostly neuro-infection, is the most common cause of not only CSE but also increased relative risk for RSE, unlike existing literature [9,10]. More common diseases such as cerebral malaria, dengue infection of the central nervous system (CNS), and typhoid encephalopathy are hardly responsible for SE in Odisha compared to Africa [11].

In this study, it was found that children less than five years of age comprised the majority of the cases (63.8%). Other studies have also found a higher prevalence in the younger age group [12,13]. As the first episode of convulsion has been theorized to be due to the underdeveloped mechanisms for control of seizure activity, there is a disruption of neuronal function with minimal abnormalities in younger children [1]. Also, younger age is more vulnerable to acute etiologies including febrile seizures. Of the acute symptomatic etiology, febrile seizures and cerebrospinal-vascular disease are the predominant cause of SE in developed countries [1,14], whereas in developing countries, CNS infection accounts for 28-47% of the etiological spectrum [15,16]. Acute symptomatic etiology (other than febrile seizures) was found to be the commonest etiology of SE in children younger than five years, and remote symptomatic etiology was common in children aged 3-10 years. This may be because of the high proportion of children with a history of prior seizures in the older age group.

A significantly high incidence of RSE with acute symptomatic etiology was found in the present study. Risk factors for RSE are sepsis, shock at presentation, pre-existing neurological abnormalities, and lag time for receiving the first AED. Children with RSE had a significantly prolonged length of stay in hospital. In this study, a less number (n = 11 [11.70%]) of patients had residual morbidity such as focal neurological deficits and neurocognitive impairment compared to another study [17,18].

A requirement of mechanical ventilation was relatively higher in the present study because most patients referred to our hospital were in critical condition and refractory status. A similar finding was reported in a study from south India [17]. The common indications for putting on the ventilator were Glasgow Coma Scale (GCS) < 7 followed by refractory shock and respiratory failure. The median (IQR) hospital stay was 7 (5-8) days, with a range of 4 to 20 days. Need for prolonged stay might be due to admission to ICU and longer time taken for neurological recovery. Similar durations of stay were reported for other diseases previously [19,20].

The mortality rate in the present study was similar to other Indian studies [21,22], whereas studies from developed countries reported comparatively less mortality (9-24%) [23,24]. The risk factors for mortality in the present study included longer duration of SE, acute symptomatic etiology, delay in receiving first AED, RSE, the requirement of ventilator support, and circulatory impairment. Transportation from longer distance led to considerable delay in starting treatment and onset of SE. We have found that acute causes of SE trend toward the worse outcome. Duration of SE is the only potentially modiﬁable determinant of mortality [21]. Therefore, besides improvisation of peripheral institutes in critical care, need for high-end ambulances to transport children with SE should be the futuristic goal of the government.

Limitations

Few causes, such as the inborn error of metabolism, autoimmune encephalitis, specific and viral encephalitis, could not be specified due to lack of investigations.

## Conclusions

Acute symptomatic etiology is the commonest cause of SE, predominantly with neuro-infections. Longer duration of SE, more lag time for receiving first AED, RSE, ventilator requirement, and presence of shock are independent predictors for poor outcomes. Upgradation of intensive care facilities even in district headquarter hospitals might improve outcomes of SE.
